# The presence of depressive symptoms and cognitive performance among older individuals with and without self-reported chronic diseases

**DOI:** 10.1590/1980-5764-DN-2023-0035

**Published:** 2023-12-04

**Authors:** Thaís Bento Lima da Silva, Tiago Nascimento Ordonez, Guilherme Alves da Silva, Maria Antônia Antunes de Souza, Sabrina Aparecida da Silva, Gabriela dos Santos, Beatriz Aparecida Ozello Gutierrez, Ana Paula Bagli Moreira, Laydiane Alves Costa, Luiz Carlos de Moraes, Patrícia Prata Lessa, Neide Pereira Cardoso, Mitsuru Sakaguchi, Henrique Salmazo da Silva, Sonia Maria Dozzi Brucki

**Affiliations:** 1 Universidade de São Paulo, School of Arts, Science and Humanities, São Paulo SP, Brazil.; 2 Universidade de São Paulo, School of Medicine, Group of Cognitive and Behavioral Neurology, São Paulo SP, Brazil.; 3 Instituto Supera de Educação, São José dos Campos SP, Brazil.; 4 Universidade Católica de Brasília, Brasília DF, Brazil.

**Keywords:** Chronic Disease, Cognition, Depression, Aged, COVID-19, Doença Crônica, Cognição, Depressão, Idoso, COVID-19

## Abstract

Dementias secondary to cardiovascular diseases are the second cause of neurogenerative diseases. These conditions can be prevented by controlling risk factors, and it is necessary to observe the relationship between chronic diseases.

**Objective:**

to know the influence of chronic non-communicable diseases on cognition and depressive symptoms in the elderly, amid the COVID-19 pandemic.

**Methods:**

578 older adults were evaluated using a sociodemographic questionnaire, the Brazilian Telephone version of the Mini Mental State Examination (Braztel-MMSE), the Geriatric Depression Scale (GDS-15) and an open questionnaire related to NCDs.

**Results:**

the association of Non-Communicable Diseases (NCD) with age, depressive symptoms and schooling was confirmed.

**Conclusion:**

no association with cognitive decline was evident due to the relationship of high schooling of participants and control of NCDs.

## RESUMO.

As demências secundárias às doenças cardiovasculares são a segunda causa de doenças neurodegenerativas. Essas condições podem ser prevenidas pelo controle de fatores de risco, sendo necessário observar a relação entre doenças crônicas.

**Objetivo:**

Conhecer a influência das doenças crônicas não transmissíveis (DCNT) na cognição e nos sintomas depressivos em pessoas idosas, em meio à pandemia da COVID-19.

**Métodos:**

Foram avaliados 578 idosos utilizando um questionário sociodemográfico, o *Brazilian telephone version of the Mini Mental State Examination* (Braztel-MMSE), a Escala de Depressão Geriátrica (GDS-15) e um questionário aberto relacionado às DCNT.

**Resultados:**

Foi confirmada a associação das DCNT crônicas com idade, sintomas depressivos e escolaridade.

**Conclusão:**

Nenhuma associação com declínio cognitivo foi evidente em razão da relação da alta escolaridade dos participantes com o controle das DCNT.

## INTRODUCTION

Aging is a natural process that is associated with molecular and cellular changes that can influence susceptibility to various chronic diseases, including neurodegenerative diseases such as Alzheimer’s disease. In a recent study by Gonzales et al.^1^, important insights were found regarding these biological alterations and how they can affect the health of older adults. The study highlights the importance of interdisciplinary approaches between gerontology, geriatrics, and neuroscience in treating chronic illnesses, including Alzheimer’s disease, which may share similar biological mechanisms. Therefore, it is important to recognize the complexity of the cellular and molecular changes that occur during aging, and how they impact the health of older adults. In this context, chronic illnesses can alter the course of a person’s life, causing changes in social roles and responsibilities, as well as impacting physical and cognitive health, affecting social activities and interactions. Changes of this type can undermine a person’s sense of autonomy, independence, and security, resulting in depressive symptoms^2^.

In 2021, Lu et al.^3^ investigated whether depressive symptoms mediated the relationship between chronic diseases and cognitive decline in older adults. They used data from the Health and Retirement Study in the United States, and employed multilevel path analysis to estimate the extent to which depressive symptoms mediated the total effect of a chronic disease on cognition. The results showed that the presence of chronic diseases, such as stroke, high blood pressure, diabetes, heart problems, and comorbidity, was associated with lower levels of cognition in both men and women, as well as lung disease in women.

In Brazil, 13% of the population is aged 60 years or older, a figure set to rise to 29% by 2050^4^. This population aging will be accompanied by a higher prevalence of chronic and cognitive diseases in older age, potentially posing a major public health problem, requiring planning of public policies for the prevention and control of non-transmissible chronic diseases (NCDs).

According to the World Health Organization, chronic NCDs are more prevalent in older people^5^. In 2008, there were approximately 36 million deaths worldwide, 63% from chronic NCDs^6^. This scenario encompasses neurodegenerative diseases, also prevalent in this contingent of the population, such as Alzheimer Disease (AD). Hence, information on tackling chronic disease and maintaining cognitive health among older adults is vital.

Currently, regarding the relationship between chronic bone diseases and dementia, it is unclear whether these diseases are a cause of cognitive decline or vice versa. However, an analysis of physiopathological aspects of these comorbidities and neurodegenerative diseases reveals some similarities in terms of risk factors and certain symptoms, such as sedentarism, vitamin D deficiency and chronic inflammation. These similarities suggest that cognitive decline and loss of bone mass are co-occurring conditions^7^.

Dementias secondary to cardiovascular diseases are the second-leading cause of neurogenerative diseases^8^. These conditions can be prevented or delayed by controlling risk factors. In this respect, the Brazilian Society of Cardiologists (SBC) states that stroke is the leading cause of mortality in Brazil, where arterial hypertension is the underlying cause of 40% of these deaths.

In this context, the primary objective of the present study was to explore the influence of self-reported NCD in older individuals amid the pandemic. More specifically, the investigation sought to collect information on sociodemographic variables, depressive symptoms and cognitive performance of participants, and determine whether any differences exist between older people with and without NCD for these variables.

## METHODS

An exploratory quantitative study was conducted. A total of 578 older individuals were screened, and expressed interest in taking part in the cognitive training study between February and September 2021. The selected individuals resided in the city of São Paulo, and were participants of the Coexistence Centers for the Elderly, or Associations of Retirees and Pensioners. Recruitment took place through the dissemination of the main study in the previously mentioned places and expression of interest by volunteers.

### Procedure

Cognitive screening assessments were performed by Gerontology graduates of the Escola de Artes, Ciências e Humanidades (EACH-USP), duly trained and supervised by the lead researchers of the study-lecturers at the EACH-USP.

The 90-minute assessment on neuropsychological variables was carried out by neuropsychologists of the Cognitive and Behavioral Neurology Research Group of the Hospital das Clínicas da Faculdade de Medicina, from Universidade de São Paulo,duly trained for the present study.

All participants signed a Free and Informed Consent Form providing guarantee of anonymity and secrecy of data, and stating the right to withdraw from the study at any time. Evaluations were conducted by telephone in previously arranged calls, with the older individuals agreeing to take part in the study. The screening process entailed assessment of 578 older adults. The invitations were extended to users of the Associations of the Third Age, Community Centers and Associations of Retirees.

### Ethical aspects

The present study was approved by the Ethics Committee for Research involving Humans of the Hospital das Clínicas da Faculdade de Medicina da Universidade São Paulo (HC-FMUSP), under approval no. 4.357.429.

### Instruments

Sociodemographic and health status questionnaire: this instrument was applied to collect the sociodemographic and health-related information of participants based on 9 questions probing self-reported chronic NCDs. Socioeconomic level was estimated using the Brazilian Criteria for Economic Classification, as defined by the Brazilian Association of Market Research Institutes^9^.

Telephone version of the Mini-Mental State Exam (Braztel-MMSE): the Braztel-MMSE is an instrument translated and adapted for use in the Brazilian population based on the Mini-Mental State Exam (MMSE). The Braztel-MMSE is a 22-item instrument with a cut-off score of 15 points. Thus, participants who scored less than 15 points were excluded from the present study^10^.

The Geriatric Depression Scale (GDS-15): this scale is designed to quantify depressive symptoms in the older population. The instrument comprises 15 questions with dichotomous responses (Yes/No). For scoring on the scale, <6 points indicates normal cognition; 6-10, mild-to-moderate depression; and >10 indicates severe depression^11^.

### Statistical analyses

We assess the relationship between the presence of chronic NCDs (dependent variable) and sex, age, education, marital status, depressive symptoms, and cognitive screening (independent variables). The sample profile was summarized in tables showing frequency and descriptive statistics expressed as location and dispersion measures, together with absolute and relative frequencies. The Kolmogorov-Smirnov test confirmed the absence of a normal distribution, and, therefore, non-parametric tests were applied. The chi-square test was used to compare categorical variables for the diagnostic groups, the *Mann-Whitney* U-test was applied to compare continuous and ordinal data, while the relationship between continuous and ordinal data was analyzed using the Spearman Correlation test.

Finally, Multiple Logistic Regression, a machine-learning method widely used for solving binary classification problems by estimating the likelihood of a given class to occur, was used in this study. The Stepwise method, a variables selection technique for reducing the number of variables in the model, was chosen to prevent overfitting and help select the optimal variables associated with chronic NCDs or otherwise. Thus, the Stepwise Multiple Logistic Regression was performed to estimate the likelihood of belonging to a given group based on the different variables of interest, such as age, sex, education, and scores on GDS15 and Braztel, employing the Stepwise technique to select the most important variables thereby precluding the inclusion of irrelevant variables in the model^12^.

Data were keyed into Google Forms and stored on Google Sheets. All statistical analyses were carried out using the JASP computer software program^12^. The level of significance (null hypothesis rejection) adopted for statistical tests was 5%, i.e., a p<0.05.

## RESULTS

A total of 578 older adults aged 60-90 years (mean 67.73±5.65 years) were assessed. Overall, participants were predominantly female, married and had mean education of 15.17±3.75 years. Participants were stratified into 2 groups according to chronic NCDs (Without NCD Group: no self-reported chronic NCDs at screening); and With NCD Group: ≥1 self-reported NCD. Statistically significant group differences were detected for age and education. However, groups proved similar for sex and marital status (Table 1). Regarding self-reported NCDs, participants reported having, on average, 1.78±1.02 chronic NCDs. Of 438 participants that reported having ≥1 NCD, 9 diseases were cited: hypertension (41%), arthritis/rheumatism (26%), lung disease (26%), diabetes mellitus (16%), osteoporosis (13%), depression (13%), cardiovascular disease (12%), malignant tumor/cancer (5%), and stroke (2%).

**Table 1:** Sociodemographic variables for overall sample and by group.

Variable		Overall		Without NCD Group		With NCD Group		
		n=578	%	n=140	%	n=438	%	
Sex	Female	446	77.16	102	72.86	344	78.53	0.163*
	Male	132	22.84	38	27.14	94	21.46	
Age	Mean (SD)	67.73 (5.65)		66.33 (4.65)		68.18 (5.86)		0.002†
	Median	67.00		65.00		67.00		
	Min-Max	60.00-90.00		60.00-78.00		60.00-90.00		
Education	Mean (SD)	15.17 (3.75)		16.29 (3.46)		14.82 (3.77)		<0.001†
	Median	15.00		16.00		15.00		
	Min-Max	0.00-30.00		8.00-28.00		0.00-30.00		
Marital status	Married	280	48.44	64	45.71	216	49.31	0.493*
	Divorced	107	18.51	32	22.86	75	17.12	
	Single	108	18.69	24	17.14	84	19.18	
	Widowed	83	14.36	20	14.29	63	14.38	
Socioeconomic level	A1 (R$ 22,716.99)	22	3.81	10	6.06	12	2.91	0.100*
	A2 (R$ 22,716.99)	24	4.15	4	2.42	20	4.84	
	B1 (R$ 10,427.74)	139	24.57	44	31.43	95	23.00	
	B2 (R$ 5,449.60)	140	24.57	42	30.00	98	23.73	
	C1 (R$ 3,042.47)	107	18.51	22	13.33	85	20.58	
	C2 (R$ 1,805.91)	50	8.65	11	6.67	39	9.44	
	D (R$ 813.56)	19	3.29	6	3.64	13	3.15	
	E (R$ 813.56)	12	2.08	1	0.61	11	2.66	
	Missing	65	10.38	0	0.00	65	14.84	

Notes:

Abbreviation: NCD, Non-Transmissible Chronic Diseases.

* chi-square test

† Mann-Whitney U-test for independent samples.

For performance on the Geriatric Depression Scale (GDS-15) and Telephone Mini-Mental State Exam (Braztel-MMSE), mean scores and standard deviations were: 2.55 (2.31) and 20.27 (1.48), respectively. There were no group differences for cognitive performance, but the With NCD Group had a higher prevalence of depressive symptoms (Table 2).

**Table 2:** Depressive symptoms and cognitive performance by group.

Variable		Overall	Without NCD Group	With NCD Group	p-value
		n=578	n=140	n=438	
GDS-15	Mean (SD)	2.55 (2.31)	2.03 (2.02)	2.72 (2.38)	<0.001
	Median	2.00	1.50	2.00	
	Min-Max	0.00 – 12.00	0.00-12.00	0.00-12.00	
BRAZTEL	Mean (SD)	20.27 (1.48)	20.38 (1.33)	20.24 (1.52)	0.487
	Median	21.00	21.00	20.00	
	Min-Max	14.00 – 22.00	16.00-22.00	14.00-22.00	

Abbreviation: NCD, Non-Transmissible Chronic Diseases. Notes: p-values for Mann-Whitney U-test for independent samples.

The results of Spearman´s correlation test revealed that age and depressive symptoms were positively correlated with the number of self-reported chronic diseases. However, years of education correlated negatively with the number of NCDs (Table 3).

**Table 3:** Spearman´s Correlation matrix for continuous variables.

Variable		No. NCDs	Age	Education	S. Level	GDS15
1. Number of NCDs	Spearman´s rho	—				
	p-value	—				
2. Age	Spearman´s rho	0.198	—			
	p-value	<0.001	—			
3. Education (years)	Spearman´s rho	-0.163	-0.161	—		
	p-value	<0.001	<0.001	—		
4. Socioeconomic level	Spearman´s rho	-0.081	-0.088	0.239	—	
	p-value	0.051	0.035	<0.001	—	
5. GDS15	Spearman´s rho	0.196	0.010	-0.120	—	
	p-value	<0.001	0.806	0.004	—	
6. Braztel	Spearman´s rho	-0.034	-0.053	0.172	-0.072	—
	p-value	0.411	0.204	<0.001	0.085	—

Abbreviation: NCD, Non-Transmissible Chronic Diseases; GDS, Geriatric Depression Scale.

Lastly, the results of the logistic regression with the Stepwise method, assessing the relationship between independent variables (sex, age, education, marital status, depressive symptoms (GDS15) and cognitive screening (Braztel)) and dependent variable (presence of chronic NCDs) are given in Tables 4 and 5 below. The aim of this process was to identify the best model for predicting the presence of chronic NCDs.

**Table 4:** Summary of logistic regression model with the Stepwise method using dependent binary variable: 1=one or more NCD; 0= no NCDs, and all remaining variables were independent.

Model	Deviation	AIC	BIC	df	ΔX²	p
1	636.591	638.591	642.947	575		
2	619.821	623.821	632.533	574	16.770	<0.001
3	610.342	616.342	629.410	573	9.479	0.002
4	601.781	609.781	627.205	572	8.561	0.003

Notes:

Abbreviation: NCD, Non-Transmissible Chronic Diseases; AIC, Akaike Information Criteria; BIC, Bayesian Information Criteria; df, Degree of freedom; ΔX², delta chi-squared.

Logistic regression models were constructed using the Stepwise method with a dependent binary variable: 1=one or more NCDs; 0=no NCDs. All other variables including Age, Sex, Marital Status, Education, Socioeconomic Level, GDS15, and Braztel, were treated as independent variables.

**Table 5:** Coefficients of the logistic regression model with the Stepwise method using a dependent binary variable: 1=one or more NCD; 0= no NCDs, with all remaining variables being independent.

Model	Parameter	Estimate	Standard error	z	Wald test		
					Wald statistic	df	p
1	(Intercept)	1.145	0.097	11.763	138.367	1	<0.001
2	(Intercept)	2.879	0.459	6.276	39.382	1	<0.001
	Education (years)	-0.111	0.028	-3.956	15.646	1	<0.001
3	(Intercept)	-1.198	1.436	-0.834	0.696	1	0.404
	Education (years)	-0.103	0.029	-3.567	12.722	1	<0.001
	Age	0.059	0.020	2.963	8.780	1	0.003
4	(Intercept)	-1.685	1.462	-1.153	1.329	1	0.249
	Education (years)	-0.096	0.029	-3.271	10.698	1	0.001
	Age	0.059	0.020	2.977	8.865	1	0.003
	GDS15	0.142	0.052	2.758	7.604	1	0.006

Abbreviation: NCD, Non-Transmissible Chronic Diseases; df, degree of freedom.

The summary of the models is given in Table 4, including standard-deviation, AIC (Akaike Information Criteria), BIC (Bayesian Information Criteria), number of parameters (df),ΔX² (delta chi-squared), p-value, McFadden, Nagelkerke, Tjur and Cox & Snell coefficients. The results reveal that Model 4 is the best, with lower AIC and BIC values, indicating a good predictive ability. The model exhibited 76% accuracy and 99% sensitivity.

The coefficients for model 4 are derived from the results of the estimate for each variable included in the model (Table 5). The Estimate column contains the estimate for each variable, while Standard Error indicates the uncertainty of the estimate, and column “z” contains the Wald test value. The Wald test shows the value of inference for each variable, and column “p” contains the corresponding p-value. According to the results, model 4 includes 3 significant variables for presence of NCDs: education (p<0.001), age (p=0.003) and GDS15 (p=0.006). The negative value for years of education indicates that the higher the education, the lower the probability of self-reported NCDs. Conversely, the estimates for age and GDS15 are positive, which shows that the older the individual and greater the number of depressive symptoms, the higher the probability of reporting ≥1 NCD.

## DISCUSSION

Evidence from a previous study showed a higher prevalence of chronic NCDs in the low-educated population, attributing this finding to several factors, including socioeconomic instability, greater exposure to stressors, and poor access to guidance promoting health^13^. This relationship is corroborated by the present study, showing that the greater the number of years of education, the lower the chances of developing chronic NCDs, i.e., years of education were negatively correlated with the number of NCDs reported.

Regarding the performance on the Geriatric Depression Scale (GDS-15) and Telephone Mini-Mental State Exam (Braztel-MMSE), mean scores and standard deviations were 2.55 (2.31) and 20.27 (1.48), respectively. Although there was no difference between the With and Without NCD Groups for cognitive performance, a distinction was detected for depressive symptoms.

By contrast, a study involving the Longitudinal Study of Adult Health (*Estudo Longitudinal de Saúde do Adulto* — ELSA-Brasil) found an association of pre-hypertension and hypertension with decline in cognitive performance. More specifically, reduced verbal fluency test scores were associated with pre-hypertension, while the decline in performance on the memory test was associated with hypertension^14^. These results might be explained by the length of the study (4 years), providing a longer period of observation and follow-up of participants.

However, the results of the present study echo those of other investigations, such as a study of older adults with hypertension controlled with the use of anti-hypertensive drugs investigating whether control of the disease prevents cognitive decline. The study in question failed to detect any significant changes^6^.

The present study showed an association between depressive symptoms and NCDs. This conclusion was based on Spearman´s correlation test, which revealed that age and depressive symptoms of participants were positively correlated with the number of self-reported chronic diseases. Nevertheless, depressive symptoms should not be regarded as a normal event of aging, but rather classified as a sign of a pathological process, which, therefore, requires adequate follow-up measures and treatment.

Negative self-perception in older hypertense adults with depressive symptoms is correlated with negative scores for quality of life^15^. Self-perceived health represents an important indicator in the life of the individual, involving physical, cognitive and emotional aspects^16^.

In a study conducted by Lu et al.^3^, the total effects of chronic diseases on cognition were partially mediated through depressive symptoms, which mediated approximately 19 to 39%, and 23 to 54%, of the total effects of chronic diseases on cognition in men and women, respectively. The authors concluded that, to understand the cognitive challenges faced by chronically ill older adults, practitioners and policymakers should consider not just the direct symptoms related to chronic diseases, but also the often-overlooked psychological conditions faced by older adults.

Depression affects people in different age groups, and there are several associated causes. A systematic review of the literature describes that about 15% of elderly people have at least one depressive symptom. There is no consensus in the literature to the effect that there is an increase in these symptoms with age, but it is noteworthy that due to changes in social roles, in some cases, reduced social interactions, in addition to the associations found in this article, elderly people tend to belong to the group most vulnerable to depression^17^.

Early diagnosis of NCDs is pivotal to the delivery of timely, effective treatment and the promotion of patient health and quality of life. Qualified professionals can control and prevent NCDs and depressive symptoms in the elderly. In this sense, public policies to encourage preventive health education, conducted by qualified professionals, can contribute to maintaining the health of the elderly, generating impacts on the prevalence and control of NCDs, in addition to symptoms associated with depression. Among these professionals, the gerontologist, due to multi-mentional training focused on the aging process and old age, can collaborate with actions aimed at prevention and health promotion for the elderly.

In summary, the present study confirmed the association of chronic NCDs with age, depressive symptoms and education. Moreover, the results reiterate the vital role of health professionals in the treatment and prevention of these diseases. Early detection is fundamental to ensure effective treatment and a healthier quality of life for older people.

Taken together, these results show that, owing to the high educational level of participants and control of NCDs, no association with cognitive decline was evident, underscoring the importance of education as a protective factor. Drawing on the arguments discussed, early diagnosis and effective treatment of depressive symptoms and hypertension confers a greater chance of preventing cognitive deficits.

The limitations of the present study include the relatively high-educated sample of the Brazilian older population, reducing generalization of its findings. Further studies encompassing different socioeconomic and educational levels should be carried out to explore the association of chronic NCDs with cognitive performance and depressive symptoms in older adults.

Moreover, future investigations involving the same methodology employed in the present investigation and representative samples of Brazilian older people in epidemiological and longitudinal studies are warranted.
